# Functional analysis of *ZmG6PE* reveals its role in responses to low-phosphorus stress and regulation of grain yield in maize

**DOI:** 10.3389/fpls.2023.1286699

**Published:** 2023-11-09

**Authors:** Hongkai Zhang, Bowen Luo, Jin Liu, Xinwu Jin, Haiying Zhang, Haixu Zhong, Binyang Li, Hongmei Hu, Yikai Wang, Asif Ali, Asad Riaz, Javed Hussain Sahito, Muhammad Zafar Iqbal, Xiao Zhang, Dan Liu, Ling Wu, Duojiang Gao, Shiqiang Gao, Shunzong Su, Shibin Gao

**Affiliations:** ^1^ State Key Laboratory of Crop Gene Exploration and Utilization in Southwest China, Sichuan Agricultural University, Chengdu, Sichuan, China; ^2^ Maize Research Institute, Sichuan Agricultural University, Chengdu, Sichuan, China; ^3^ Key Laboratory of Biology and Genetic Improvement of Maize in Southwest Region, Ministry of Agriculture, Chengdu, Sichuan, China; ^4^ Centre of Excellence for Plant Success in Nature and Agriculture, The Queensland Alliance for Agriculture and Food Innovation (QAAFI), The University of Queensland, St. Lucia, Brisbane, QLD, Australia; ^5^ Key Laboratory of Wheat and Maize Crops Science, College of Agronomy, Henan Agricultural University, Zhengzhou, China

**Keywords:** maize, low-phosphorus stress, ZmG6PE, transcriptomics, metabonomics

## Abstract

A previous metabolomic and genome-wide association analysis of maize screened a glucose-6-phosphate 1-epimerase (*ZmG6PE*) gene, which responds to low-phosphorus (LP) stress and regulates yield in maize’s recombinant inbred lines (RILs). However, the relationship of *ZmG6PE* with phosphorus and yield remained elusive. This study aimed to elucidate the underlying response mechanism of the *ZmG6PE* gene to LP stress and its consequential impact on maize yield. The analysis indicated that ZmG6PE required the Aldose_epim conserved domain to maintain enzyme activity and localized in the nucleus and cell membrane. The *zmg6pe* mutants showed decreased biomass and sugar contents but had increased starch content in leaves under LP stress conditions. Combined transcriptome and metabolome analysis showed that LP stress activated plant immune regulation in response to the LP stress through carbon metabolism, amino acid metabolism, and fatty acid metabolism. Notably, LP stress significantly reduced the synthesis of glucose-1-phosphate, mannose-6-phosphate, and β-alanine-related metabolites and changed the expression of related genes. *ZmG6PE* regulates LP stress by mediating the expression of *ZmSPX6* and *ZmPHT1.13*. Overall, this study revealed that *ZmG6PE* affected the number of grains per ear, ear thickness, and ear weight under LP stress, indicating that *ZmG6PE* participates in the phosphate signaling pathway and affects maize yield-related traits through balancing carbohydrates homeostasis.

## 1 Introduction

Maize (*Zea mays* ssp. *mays*) is a globally imperative food, feed, and industrial feedstock crop (Niu et al., 2020). Growth of maize requires a substantial amount of phosphorus-based fertilizer, but it is a finite and non-renewable resource. It is concerning that phosphate rock is projected to be depleted within the next 100 years (Kochian, 2012). Roots absorb the phosphorus mainly in the form of inorganic phosphate (Pi), but microorganisms and metal cations quickly convert it into unavailable forms by precipitation and fixation (Liu et al., 2005; Chiou and Lin, 2011; Ham et al., 2018). Therefore, determining how to reduce the amount of phosphorus fertilizer while maintaining a high crop yield is an important problem deserving further research.

Plants have evolved complex and sophisticated regulatory systems to adjust to a varying soil phosphate environment to maintain growth. Phosphate starvation-induced (*PSI*) genes are mainly regulated by Phosphate Starvation Response-SYG1/PH081/XPRI (PHR-SPX) modules that play key roles in this network (Puga et al., 2014; Wang et al., 2018). Phosphate Starvation Response (PHR) transcription factors (a sub-family of MYB TFs) positively regulate *PSI* gene expressions by attaching to PHR1 Binding Sequence (P1BS) (Rubio et al., 2001; Zhou et al., 2008). The activities of PHRs are balanced post-transcriptionally by negatively regulating TFs known as SPX-domains containing proteins through recognizing soluble inositol polyphosphates (InsPs) levels in cytosol and preventing the binding of PHRs to P1BS (Wang et al., 2014; Wild et al., 2016). Phosphate Starvation Response (PHR) transcription factors induced the transcription of plasma membrane-localized phosphate transporter (PHT), which is a class of phosphorous transporters playing crucial roles in phosphate uptake/distribution/redistribution in plants and enhances the transmembrane transport of phosphate under LP stress (Gonzalez et al., 2005; Sun et al., 2012). By elucidating the mechanism of phosphorus tolerance, it is possible to effectively improve maize yield while reducing the application of phosphorous fertilizer.

Previous studies have shown that LP stress activates physiological responses in plants, leading to changes in the levels of related metabolites. Under phosphate deficiency stress, plant pile up starch and sugar, which promotes carbon allocation to the root system and facilitates further soil phosphorus excavation. Low-phosphorus (LP) stress affects starch metabolism, inhibits photosynthesis in crop plants, and triggers starch accumulation in leaves (Gibson, 2004; Morcuende et al., 2007). Sucrose upregulated the activities of D-glucuronic acid, 5-O-methylembelin, and N-acetyl-L-phenylalanine during LP conditions (Yang et al., 2020). A metabolome analysis showed that LP stress accelerated glycolysis in the roots of soybean and repressed malic acid synthesis (Li et al., 2022). Under LP conditions, LP-tolerant maize genotypes induce the transcription of genes regulating plant hormone signaling, acid phosphatase, and metabolite, which promote phosphorus absorption and utilization in maize (Jiang et al., 2017).

Metabolomic profiling was conducted on six LP tolerant and sensitive maize inbred lines, and combined with genome-wide association analysis, five genes were identified to be responsive to LP stress. Among them, *ZmG6PE* not only influenced phosphorus content but also responded to LP stress by affecting ear diameter and ear rows (Luo et al., 2019). Glucose‐6‐phosphate 1‐epimerase catalyzes the conversion of anomeric forms of α-D-glucose‐6‐phosphate to β-D-glucose‐6‐phosphate at the branch point of D-glucose metabolism (Wurster and Hess, 1972; Wurster and Hess, 1973; Graille et al., 2006). This enzyme is named YMR099C in *Saccharomyces cerevisiae*, and it has an active site with sulfate ion connected by an arginine clamp formed by a side chain of two highly conserved residues of arginine (Graille et al., 2006). A bioinformatics analysis of the budding yeast’s entire genome for finding the putative target of transcription factor “GCN4 (a positive transcription factor in yeast, binds general control promoters at all 5' TGACTC 3' sequences)” has shown that GCN4 regulates YMR099C (Schuldiner et al., 1998). Trz1 (a tRNA 3' end processing endonuclease), Nuc1 (a mitochondrial nuclease), and YMR099C form highly stable heterohexamers consisting of two copies of each of the three subunits, which suggests that YMR099C and Trz1 may regulate apoptotic nucleases activity (Ma et al., 2017). Glucose-6-phosphate is an important metabolite in multiple metabolic pathways, such as glycolysis and the pentose phosphate pathway (Wu et al., 2018; He et al., 2021). Glycolysis is a metabolic precursor for producing biomass and supplies cellular energy (Tanner et al., 2018). The pentose phosphate pathway can additionally catalyze the production of nicotinamide adenine dinucleotide phosphate (NADPH) using glucose-6-phosphate dehydrogenase (G6PDH) for providing reductive power (Jiang et al., 2012; Fouquerel et al., 2014). Currently, research on glucose-6-phosphate 1-epimerase mainly focuses on microorganisms and has not been studied in plants (Wurster and Hess, 1972; Wurster and Hess, 1973; Graille et al., 2006). Therefore, the current study would be a reference for investigating the future study of glucose-6-phosphate-1 epimerase in plants.

In sum, ZmG6PE is a glucose-6-phosphate 1-epimerase that regulates carbohydrate homeostasis. ZmG6PE is an enzyme that catalyzes α-D-glucose‐6‐phosphate to β-D-glucose‐6‐phosphate and participates in glycolysis and the pentose phosphate pathway. By biochemical, omics, molecular biology, and genetics analysis, the present study found that the gene function of *ZmG6PE* affects maize yield and the mechanism of response to LP stress. This research provides new directions for the improvement of maize yield and the study of LP tolerance mechanisms.

## 2 Material and methods

### 2.1 Plant materials and growth conditions

Using the CRISPR/Cas9 (Clustered regularly interspaced short palindromic repeats/CRISPR-associated protein 9) system, the *ZmG6PE* gene was knocked out in the maize inbred line KN5585 by the *Agrobacterium tumefaciens*-mediated transformation method. The mutation site was detected using ZmG6PE-KO-F and R primers (**Supplementary Table S1**). Maize Research Institute of Sichuan Agricultural University provided the seeds of maize inbred lines 178 and 9782 for the current study.

Seeds were surface disinfected with 2% (V/V) sodium hypochlorite (NaClO) solution for 30 min, then rinsed with ddH_2_O 5-6 times to remove the NaClO solution and were germinated on filter paper in dark conditions at 28 °C for 3 days. Germinated seeds were cultivated in sand and gravel for 7 days, and the seedlings with consistent growth were selected to remove embryos and endosperms. The seedlings were then transferred to plastic containers containing 25 L of half Hoagland nutrient solution concentration for adaptation cultivation for 3 days. Subsequently, the nutrient solution was replaced with normal-phosphorus (NP) (1 mM) or LP (1 μM) solution, also containing 6 mM KNO_3_, 4 mM Ca (NO_3_)_2_.4H_2_O, 1 mM or 1 μM NH_4_H_2_PO_4_, 100 μM EDTA-Fe, 2 mM MgSO_4_.7H_2_O, 46 μM H_3_BO_3_, 0.146 μM MnCl_2_.4H_2_O, 0.76 μM ZnSO_4_.7H_2_O, 0.016 μM (NH4)_6_Mo_7_O_24_.4H_2_O, and 0.32 μM CuSO_4_.5H_2_O, with a pH of 5.5 (Luo et al., 2019). The nutrient solution was replaced every three days, and the ventilation pump was operated for 8 hours every day.

### 2.2 Reverse transcription-quantitative polymerase chain reaction (RT-qPCR) analysis

The total RNA was extracted using a Trizol reagent kit (Invitrogen, Thermo Fisher Scientific, Waltham, MA, USA) according to manufacturer’s instructions. The reverse transcription of the first strand was performed using the PrimeScript™ II 1^st^ Strand cDNA synthesis kit by following the manual’s instructions. The RT-qPCR was carried out using the FastStart Essential DNA Green Master (Roche, Germany) on a CFX96 Real-Time PCR Detection System (Bio-Rad, USA) following the kit’s protocol. The total reaction volume consisted of 5 μl 2× FastStart Essential DNA Green Master, with 0.5 μl each of forward and reverse primers and 1 μl of the cDNA template. The amplification procedure was set as follows; initial denaturation at 95 °C for 10 min; then 40 cycles of 95 °C for 5 sec, 60 °C for 15 sec, and 72 °C for 20 sec; and finally, a melting curve process. The quantitative PCR primers were designed using Beacon Designer™ 7 software and shown in **Supplementary Table S1**. All experiments were conducted thrice with three technical replicates.

### 2.3 Total phosphorus content measurements in plants

Approximately 0.2 g of dry tissues were digested using H_2_SO_4_-H_2_O_2_ at 420 °C to obtain the total phosphorus. Then, phosphorus in the supernatant was measured using Auto Discrete Analyzers (SmartChem 200).

### 2.4 Measurements of starch and soluble sugar

A starch content kit (Code: G0507W, Grace Biotechnology Co., LTD, Suzhou, China) was used to assay the starch content of maize leaves. The soluble sugar content of maize leaves was determined by a plant soluble sugar content kit (Code: KT-1-Y, Comin Biotechnology Co., LTD, Suzhou, China). The determination of the starch and soluble sugar was carried out strictly according to the operation instructions of the kit.

### 2.5 Purification and activity analysis of ZmG6PE

The full-length coding sequence of *ZmG6PE* was introduced into the pCold vector and then transformed into Rosetta-competent cells. The optimal protein induction conditions were screened, and the culture broth was scaled up under the optimal conditions. The extracted protein was purified and stored at -80 °C for future use.

Kinetic reactions were carried out at 25 °C in 50 mM KCl, 50 mM imidazole, and 8 mM MgSO_4_ at pH 7.6. The absorbance values were measured at 340 nm using the SW18-MV Stopped Flow Spectrophotometer (Applied Photophysics, UK). Reactions were started by mixing equivalent volumes of equilibrated 2 mM nicotinamide adenine dinucleotide phosphate (NADP^+^) and 60 μM glucose-6-phosphate with 160 units/mL glucose-6-phosphate dehydrogenase from *Leuconostoc mesenteroides* (Sigma) and the varying concentrations of ZmG6PE (Graille et al., 2006).

### 2.6 RNA-seq analysis

For transcriptome analysis, the second fully expanded leaves and the roots of the mutant *zmg6pe* and wild type (WT) plants were harvested and frozen in liquid nitrogen immediately. The Trizol reagent (Invitrogen Life Technologies) was used to isolate total RNA from the leaves and roots following the manual’s instructions. RNA’s concentration, integrity, and quality were measured and assessed using a NanoDrop™ spectrophotometer (Thermo Scientific). RNA samples were prepared using 3 μg RNA as the initial input material. The RNA-seq and data analysis were carried out by the Shanghai Bioprofile Biotechnology Co., Ltd. (Shanghai, China). The sequencing libraries of the total RNA were constructed using the TruSeq RNA Sample Preparation Kit (Illumina, San Diego, CA, USA), and the RNA libraries were sequenced by the Illumina NovaSeq 6000 platform. Each sample consists of three plants with three replicates.

### 2.7 Metabolite analysis

For extracting the metabolites, samples were weighed, dried, and transferred into a 1.5 mL Eppendorf tube containing a 5 mm tungsten bead and grounded in a grinding mill for 1 min with 65 Hz power. Metabolite extraction was performed using an ultrasonic shaker with 1 mL precooled acetonitrile, methanol, and H_2_O (v:v:v, 2:2:1) at 4 °C for 1 hour. The mixture was placed at -20 °C for 1 hour and then centrifuged at 14,000 rpm for 20 min at 4 °C. The supernatants were collected and placed in a cryogenic vacuum for concentration. Subsequently, the UPLC-ESI-Q-Orbitrap-MS system (UHPLC, Shimadzu Nexera X2 LC-30AD, Shimadzu, Japan) coupled with Q-Exactive Plus (Thermo Scientific, San Jose, CA, USA) was used for Metabolomic profiling.

The MS-DIAL software was used to process the raw MS data for retention time correction, peak alignment, and peak area extraction. The accuracy mass (mass tolerance < 0.02 Da) and MS/MS data (mass tolerance < 0.02 Da) were matched with Kyoto Encyclopedia of Genes and Genomes (KEGG), MassBank, and other available databases, and a standard self-built metabolite library was used to differentiate and recognize the metabolites. Only variables greater than 50% non-zero measured values within at least 1 group were processed in extracted ion features. The samples used in the metabolite analysis were consistent with those used in the RNA-seq analysis.

### 2.8 Subcellular localization of ZmG6PE

To localize ZmG6PE protein in the cell, a full-length coding sequence of *ZmG6PE* was introduced into pCAMBIA2300-35S-eGFP using the NovoRec^®^ plus One Step PCR Cloning Kit (NovoProtein, Shanghai, China). The constructs were mobilized into an *Agrobacterium* strain GV3101 by the freeze-thaw method and cultured for up to the required density. Then cells were collected and resuspended in a solution comprising 10 mM magnesium chloride and 150 mM acetosyringone (Sigma-Aldrich). Fluorescence signals of the eGFP in *Nicotiana benthamiana* leaves were detected with a confocal microscope (Leica).

## 3 Results

### 3.1 ZmG6PE is localized in the nucleus and cell membrane

**Figure 1:**
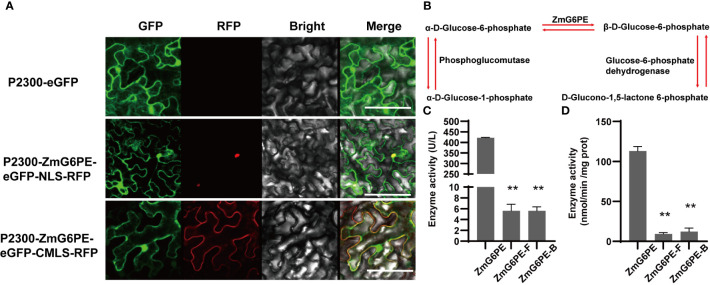
Subcellular localization of ZmG6PE and enzyme activity. **(A)** Subcellular localization of ZmG6PE in tobacco leaves, bar = 10 nm. **(B)** Schematic representation of enzymatic reactions involving glucose-6-phosphate anomer. **(C)** Determination of the enzyme activity by the enzyme kinetic method. **(D)** Determination of enzyme activity using an ELISA kit. “**” represents p < 0.01 by single factor ANOVA test.

The subcellular localization of the ZmG6PE protein was determined by introducing the combined vector pCAMBIA2300-P35S: ZmG6PE-eGFP and the nucleus localization signal (NLS) into tobacco leaves using transient transformation. The signals derived from ZmG6PE overlapped with the autofluorescence of NLS (**Figure 1A**). Similarly, the signals derived from ZmG6PE overlapped with the autofluorescence of the cell membrane localization signal (CMLS) (**Figure 1A**). These results confirmed the prediction of CELLO v.2.5, showing that the ZmG6PE protein is localized in the nucleus and cell membrane.

### 3.2 ZmG6PE exhibits glucose-6-phosphate 1-epimerase activity

ZmG6PE protein sequence from 41 to 308 amino acids encodes a conserved domain of Aldose_epim (**Supplementary Figure S1A**). To explore the critical peptide sequences of ZmG6PE, we divided ZmG6PE into two sections, ZmG6PE-Front (ZmG6PE-F) and ZmG6PE-Behind (ZmG6PE-B). Both sections (ZmG6PE-F and ZmG6PE-B) contained the conserved Aldose_epim domains of ZmG6PE (41 to 308 aa), ZmG6PE-F (41 to 147 aa), and ZmG6PE-B (1 to 161 aa) (**Supplementary Figure S1A**). Unlike ZmG6PE-B and ZmG6PE-F, the tertiary structure of ZmG6PE had a putative active site pocket (**Supplementary Figure S1B**). To investigate the biochemical characteristics of ZmG6PE, His-tagged ZmG6PE, ZmG6PE-B, and ZmG6PE-F proteins were expressed in *Escherichia coli* (**Supplementary Figure S1C**). The enzymatic activity of protein during the interconversion of α- and β-D-glucose-6-phosphate anomers is explained by the specificity of the enzymes participating in sugar metabolism (**Figure 1B**). Enzyme kinetics and enzyme-linked immunosorbent assay (ELISA) were employed to evaluate the ZmG6PE activity. The results showed that ZmG6PE had glucose-6-phosphate 1-epimerase activity (**Figures 1C, D**); however, the ZmG6PE protein segments (ZmG6PE-F and ZmG6PE-B) exhibited negligible glucose-6-phosphate 1-epimerase activity (**Figures 1C, D**).

### 3.3 The *zmg6pe* mutant is sensitive to LP stress

**Figure 2:**
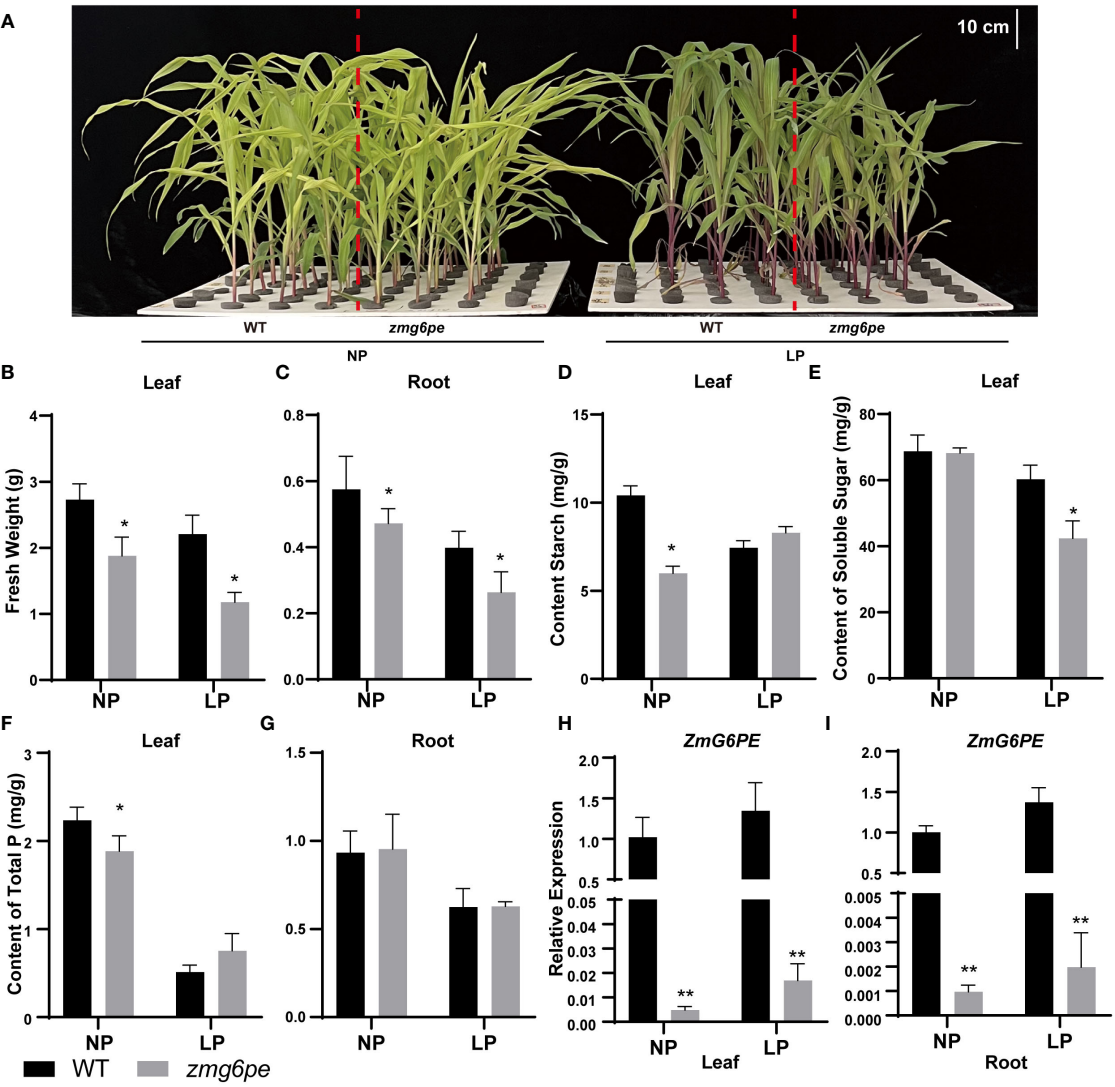
Responses of *ZmG6PE* in hydroponic culture with different phosphorous concentrations. **(A)** WT and *zmg6pe* mutants under NP and LP hydroponics. **(B, C)** Fresh weight. **(D)** Starch content. **(E)** Soluble sugar content. **(F, G)** Total phosphorus content. **(H, I)**. The expression levels of *ZmG6PE* gene. “*” represents 0.01< p < 0.05, “**” represents p < 0.01, single factor ANOVA test.

To investigate the functions of *ZmG6PE* in maize, two independent transgenic lines (*zmg6pe-1* and *zmg6pe-2*) were developed using CRISPR/Cas9. The line *zmg6pe-1* has a 421 bp deletion, while *zmg6pe-2* has a 1 bp deletion (**Supplementary Figure S2A**), thus both result in a truncated protein. During the seedling stage, the fresh weight and phosphorus content in *zmg6pe* decreased compared to WT (**Supplementary Figures S2B-D**), but there was no significant difference at maturity (**Supplementary Figures S2E-G**). To determine whether *ZmG6PE* responds to LP stress, 10 days old maize seedlings were further raised in a hydroponic system and treated with 1 mM and 1 μM phosphate conditions for 15 days (**Figure 2A** and **Supplementary Figure S2H**). Under both NP and LP conditions, the leaves and roots fresh weight of the *zmg6pe* significantly reduced (**Figures 2B, C**); LP tolerance coefficients in *zmg6pe* leaves and roots were reduced (**Supplementary Figures S2I, J**). Starch contents decreased under NP conditions in leaves of *zmg6pe* (**Figures 2D**). Soluble sugar contents decreased under LP conditions in the leaves of *zmg6pe* (**Figures 2E**). The phosphorus contents decreased under NP conditions in *zmg6pe* leaves than in WT leaves (**Figure 2F**). However, the phosphorus content in roots did not significantly change in WT and *zmg6pe* (**Figure 2G**). It significantly reduced the expression levels of *ZmG6PE* in the leaves and roots of the *zmg6pe* mutant (**Figures 2H, I**).

### 3.4 Functional analysis of the *ZmG6PE* gene in response to LP stress

The *zmg6pe* had reduced fresh weight under LP conditions, indicating that *ZmG6PE* responds to LP stress. In order to elucidate the regulatory mechanisms of *ZmG6PE* under LP conditions, comparative transcriptome analysis of leaves and roots was conducted between WT and *zmg6pe* mutant. There were 2072, 2367, and 504 overlapping differentially expressed genes (DEGs) in leaves (L), roots (R), and both in leaves and the roots (LR), respectively (**Supplementary Figure S3A**).

**Figure 3:**
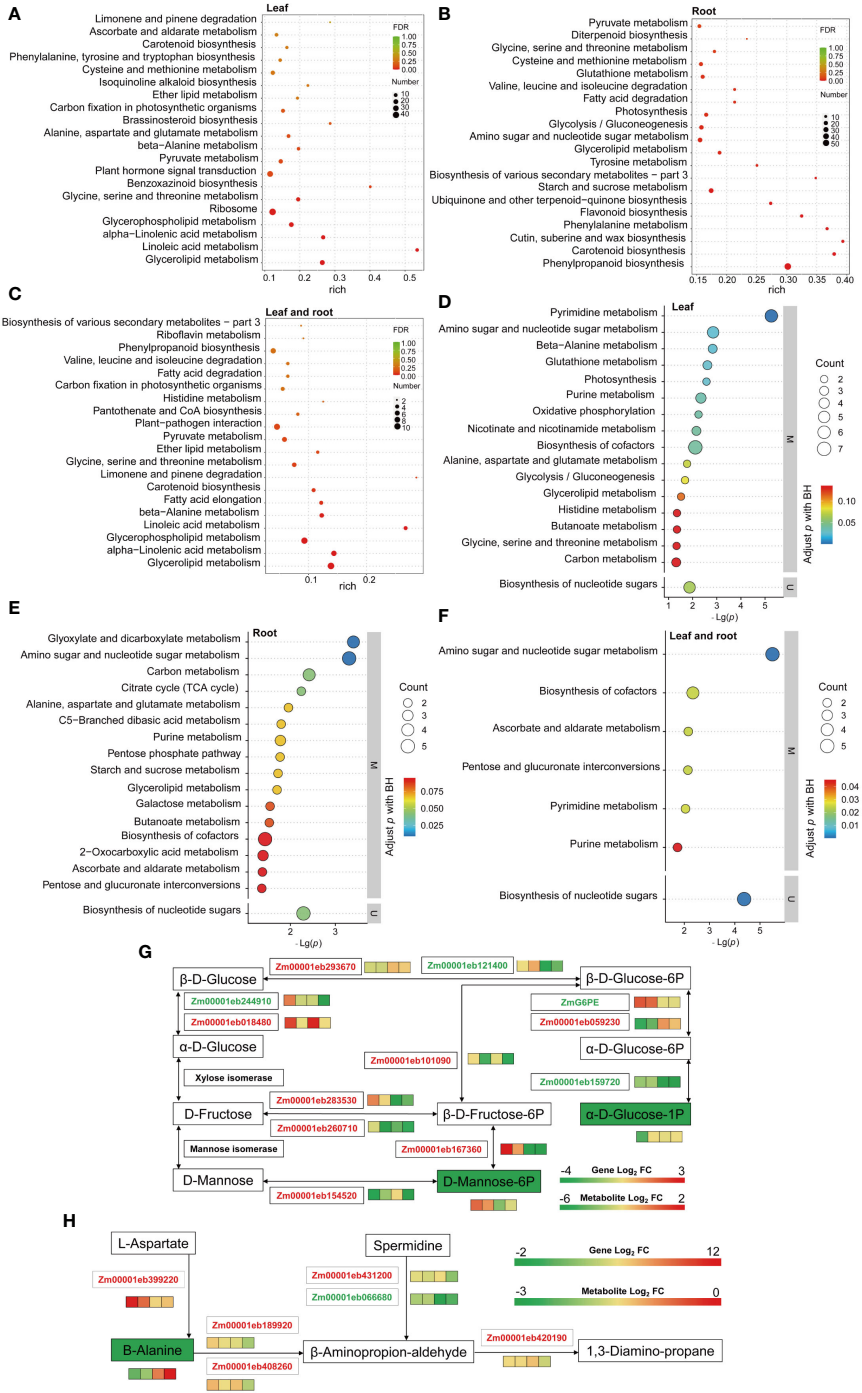
KEGG pathway enrichment analysis performed on the DEGs and DEMs, and channel heat map analysis. **(A–C)** KEGG enrichment analysis of the overlapping DEGs in leaves, roots, and shared in leaves and roots, respectively. **(D–F)** KEGG enrichment analysis of the overlapping DEMs in leaves, roots, and shared in leaves and roots, respectively. **(G)** Glycolysis/gluconeogenesis, fructose, and mannose metabolic pathways involve phosphorus transport in leaves and roots. **(H)** The β-alanine metabolism involves phosphorus transport in leaves and roots. Boxes are WT-NP-L vs. WT-LP-L, *zmg6pe*-NP-L vs. *zmg6pe*-LP-L, WT-NP-R vs. WT-LP-R, and *zmg6pe*-NP-R vs. *zmg6pe*-LP-R from left to right. Red and green fonts indicate upregulated and downregulated genes, respectively. Green and red solid squares indicate downregulated and upregulated metabolites, respectively.

KEGG pathway enrichment analysis revealed that terms related to carbon metabolism, amino acid synthesis and metabolism, fatty acid metabolomic pathway, immune regulation, and plant hormones were highly enriched in L (**Figure 3A** and **Supplementary Figure S3B**). While terms related to carbon, amino acid and fatty acid metabolisms, amino acid degradation, secondary metabolites, and vitamins were highly enriched in the R (**Figure 3B** and **Supplementary Figure S3C**); similarly, the terms related to carbon, amino acid and fatty acid metabolisms, amino acid degradation, immune regulation, and secondary metabolites were enriched in LR (**Figure 3C** and **Supplementary Figure S3D**). As such, LP stress activated the immune response system in seedlings, and *ZmG6PE* regulated carbon, amino acid and fatty acid metabolisms, plant hormones, and secondary metabolites in response to LP stress.

### 3.5 Metabolite analysis of the *ZmG6PE* gene in response to LP stress

Transcriptome KEGG enrichment analysis showed that LP treatment affected carbon and amino acid metabolisms, indicating that LP stress caused variations in the synthesis and metabolism of nitrogen- and carbon-related compounds. To further investigate the function of *ZmG6PE*, comparative metabolomic analyses were performed in *zmg6pe* and WT seedlings under LP and NP conditions. There were 164, 96, and 26 overlapping differentially expressed metabolites (DEMs) found in L, R, and LR, respectively (**Supplementary Figure S4A**). The metabolomic analysis of L and R samples revealed significant KEGG pathway enrichment in carbon metabolism, amino acid metabolism, genetic material synthesis, and immune regulation, with some more enrichments of fatty acid metabolism, enzymatic activity, and energy metabolism in L (**Figures 3D-F** and **Supplementary Figures S4B-D**). These results indicate that LP stress may activate the plant immune response system, whereas *ZmG6PE* affects LP stress by regulating the above-mentioned metabolic pathways.

### 3.6 Combined analysis revealing *ZmG6PE* involved in KEGG pathways in response to LP stress

A combined transcriptome and metabolome analysis was performed to examine whether there is any association between gene expression patterns and metabolite accumulation. Enrichment analysis revealed 42, 15, and 12 KEGG pathways in L, R, and LR, respectively (**Supplementary Figure S5A**). In the combined analysis of the KEGG pathways, the carbon metabolism, amino acid synthesis and metabolism, glycolysis/gluconeogenesis, secondary metabolite biosynthesis, and carbon fixation in photosynthesis were found enriched in L (**Supplementary Figure S5B**). In R, galactose metabolism, starch and sucrose metabolism, carotenoid metabolism, photosynthesis, and secondary metabolite biosynthesis were highly enriched (**Supplementary Figure S5C**). In LR, significant enrichment was observed in carbon, amino acid and fatty acid metabolisms, and immune regulation (**Supplementary Figure S5D**). These results indicate that LP stress may activate immune regulation and the *ZmG6PE* gene affects LP stress by regulating primary and secondary metabolism.

The combined network analysis found that α-D-glucose-1 phosphate in the glycolysis/gluconeogenesis pathway and D-mannose-6 phosphate in fructose and mannose metabolism differed significantly (**Figure 3G**). The pathway map showed significant reductions in α-D-glucose-1 phosphate and D-mannose-6 phosphate. Zm00001eb244910, Zm00001eb121400, Zm00001eb159720, and *ZmG6PE* gene expression levels decreased, while Zm00001eb293670, Zm00001eb018480, Zm00001eb059230, Zm00001eb101090, Zm00001eb283530, Zm00001eb260710, Zm00001eb154520, and Zm00001eb167360 gene expression levels increased. These results indicate that glucose-1-phosphate- and mannose-6-phosphate-related genes and metabolites respond to LP stress.

The pathway map showed significant reduction in β-alanine (**Figure 3H**). Zm00001eb066680 gene expression level decreased, while Zm00001eb399220, Zm00001eb431200, Zm00001eb189920, Zm00001eb408260, and Zm00001eb420190 gene expression levels increased. These results indicate that β-alanine genes and metabolites respond to LP stress.

### 3.7 Expression of *ZmSPX2* and *ZmPHT1.13* are down-regulated in *zmg6pe* mutant

**Figure 4:**
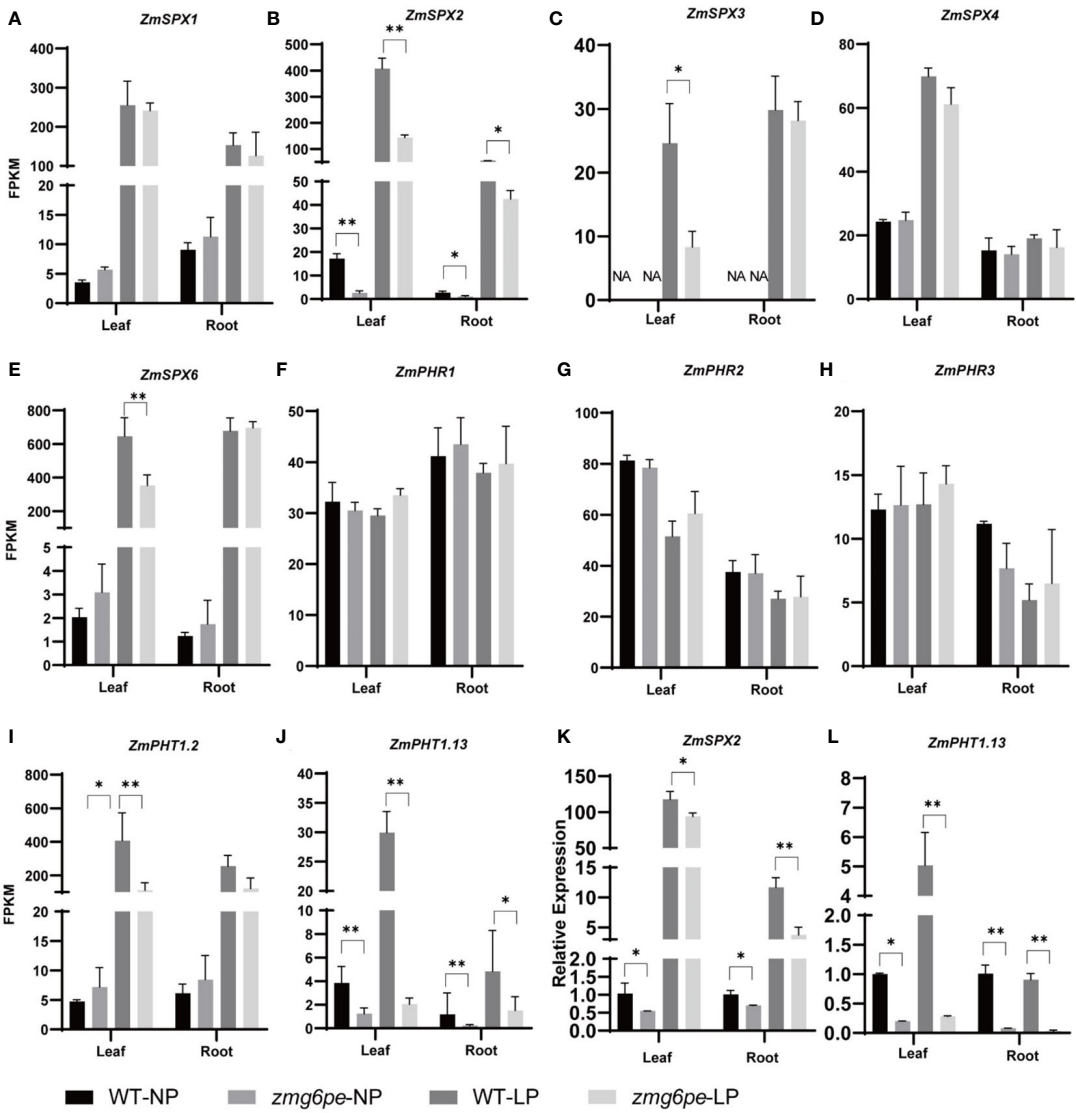
Analysis of PSI genes expression levels. **(A–J)** Expression levels (FPKM) of *ZmSPX1*, *ZmSPX2*, *ZmSPX3*, *ZmSPX4*, *ZmSPX6*, *ZmPHR1*, *ZmPHR2*, *ZmPHR3*, *ZmPHT1.2*, and *ZmPHT1.13*, respectively, in leaves and roots under NP and LP conditions. **(K, L)** Expression levels of *ZmSPX2* and *ZmPHT1.13* in leaves and roots under NP and LP, respectively, by RT-qPCR. Values are given as the mean ± SD and indicate the average FPKM values from three biological replicates of RNA-seq libraries. “*” represents p < 0.05, and ”**” represents p < 0.01 by single factor ANOVA test.

Phosphate Starvation Response (PHR) transcriptional factors are the central regulatory factors regulating the activities of LP-responsive genes in the LP regulatory network (Nilsson et al., 2007; Zhou et al., 2008; Lu et al., 2020). The phosphate transporter (PHT) mediates the absorption and transport of Pi in the plant rhizosphere (Shin et al., 2004; Sun et al., 2012). SYG1/PH081/XPRI (SPX) is a negative regulator of PHR, and regulates phosphorus transport and homeostasis in plants (Liu et al., 2010). To further explore the pathways that regulate the phosphorus signal and expression levels of *PSI* genes were investigated. Transcriptome analysis of *PSI* genes revealed that LP treatment promoted the expression of *ZmSPXs* (*ZmSPX1*, *ZmSPX2*, *ZmSPX3*, *ZmSPX4*, and *ZmSPX6*) and *ZmPHTs* (*ZmPHT1*.*2* and *ZmPHT1*.*13*), ut the expression of *ZmPHRs* (*ZmPHR1, ZmPHR2*, and *ZmPHR3*) did not show significant changes (**Figures 4A–J**). Interestingly, regardless of phosphorus status, the expression levels of *ZmSPX2* and *ZmPHT1*.*13* in *zmg6pe* were significantly lower than WT in both L and R (**Figures 4B, J**). The RT-qPCR (**Figures 4K, L**) confirmed the above results, suggesting that *ZmG6PE* is probably involved in responding to LP stress by mediating expressions of *ZmSPX2* and *ZmPHT1.13*. Under LP conditions, the lack of significant differences in phosphorus content between WT and *zmg6pe* mutants might be attributed to the enhanced expression of *ZmSPX2*, which inhibits the expression of *ZmPHRs* and *ZmPHT1.13*.

### 3.8 ZmG6PE regulates the yield-related traits of maize grains

**Figure 5:**
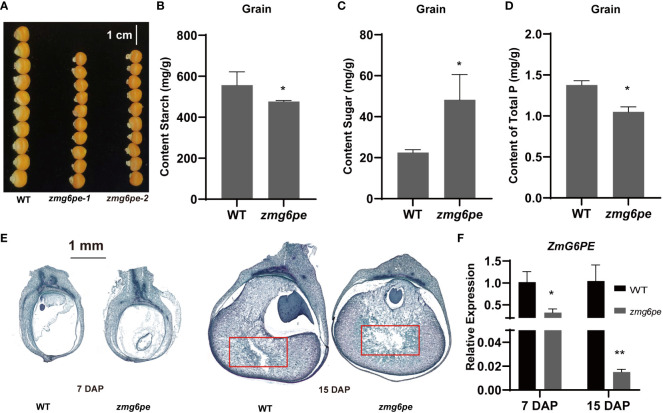
Analysis of maize kernel development. **(A)** Grain size. **(B–D)** Content of the grain’s starch, sugar, and total phosphorus, respectively. **(E)** Paraffin section of grains at 7 and 15 DAP. **(F)** The expression levels of ZmG6PE in grain were measured at 7 and 15 DAP. “*” represents p < 0.05, “**” represents p < 0.01 by single factor ANOVA test.

To assess the effects of ZmG6PE on yield-related traits, we investigated the yield-related data for 2021-Qujing (QJ) and 2022-Chongzhou (CZ) grown materials. Results showed that the ear length, ear weight, 100-grain weight, and number of grains per ear were significantly difference in the *zmg6pe* compared to WT (**Supplementary Table S2**). Besides this, the grain size in *zmg6pe* was significantly smaller (**Figure 5A**). Moreover, the sugar contents increased, and starch contents decreased in the *zmg6pe* compared to the WT (**Figures 5B, C**). The phosphate content in the *zmg6pe* was significantly lower than in WT (**Figure 5D**). The observation of paraffin sections revealed that grain filling in WT was normal at 7 days after pollination (DAP). In contrast, *zmg6pe* showed abnormal grain filling (**Figure 5E**). At 15 DAP, the starch particles in the WT seeds were fully packed, while *zmg6pe* had irregular cavities. The red box area of the *zmg6pe* had a significantly larger cavity than WT (**Figure 5E**). The *ZmG6PE* gene expression levels significantly decreased at 7 and 15 DAP in the *zmg6pe* (**Figure 5F**). The above results demonstrate that the *ZmG6PE* gene affects maize yield.

### 3.9 Biparental segregation population validation/haplotype analysis

**Figure 6:**
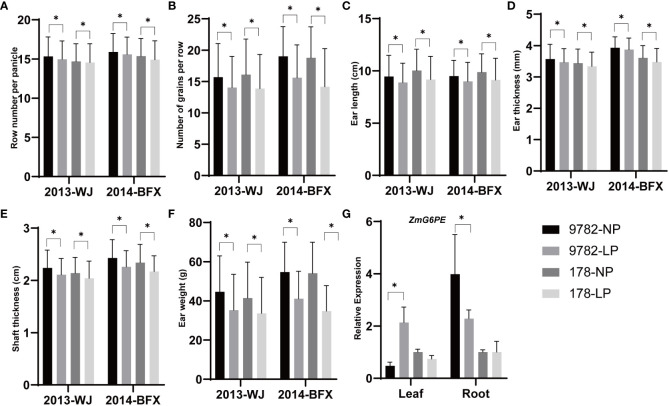
Yield-related analysis of the RILs. **(A–F)** Significantly different traits between segregating genotypes of *ZmG6PE*. WJ, Wenjiang Farm; BFX, Bifengxia Farm. **(G)** The gene expression level of *ZmG6PE*. “*” represents p < 0.05 by single factor ANOVA test.

To investigate the effects of ZmG6PE on yield under LP conditions, the yield-related traits of RILs derived from inbred lines 178 and 9782 were measured. The amplification of the *ZmG6PE* gene coding sequences in the inbred lines 178 and 9782 revealed no differences (**Supplementary Figure S6A**). However, an insertion or deletion (Indel) was detected in the promoter sequence of the *ZmG6PE* gene in two inbred lines, with a 23 bp deletion in inbred line 178 compared to inbred line 9782 (**Supplementary Figure S6B**), which could be classified into two haplotypes (178 genotypes and 9782 genotypes) (**Supplementary Figure S6C**). Genotype statistics were performed on the RILs, and the yield-related traits were researched from the Wenjiang Farm in 2013 and the Bifengxia Farm in 2014 under LP conditions. Row number per panicle, number of grains per row, ear length, ear thickness, shaft ear thickness and ear weight of genotype 178 were significantly lower than those of genotype 9782 (**Figures 6A–F**). In inbred line 9782, the expression of the *ZmG6PE* gene varied with changes in the phosphorus concentration, while in inbred line 178, gene expression was unaffected by the phosphorus concentration (**Figure 6G**). These results indicate that ZmG6PE plays a role in maize yield and phosphorus content.

## 4 Discussion

### 4.1 ZmG6PE is a key enzyme that supports life activities

Anomeric carbon is essential in glycan reactions and quickly provides metabolites for physiological processes (Gomes et al., 2015). Glucose-6-phosphate has two forms of α and β anomers (Wurster and Hess, 1972; Wurster and Hess, 1973; Graille et al., 2006). The isomerization of α-D-glucose-6-phosphate by glucose-6-phosphate isomerase produces fructose 6-phosphate (Aguilar-Pontes et al., 2018). Phosphoglucomutase catalyzes the reversible isomerization of α-D-glucose-6-phosphate to glucose-1-phosphate, which is converted into ADP-glucose (precursor of starch synthesis) by ADP-glucose-pyrophosphorylase (Geigenberger et al., 2004; Zeeman et al., 2007). Combined analysis reveals significant differences in glucose-1-phosphate levels, and starch content reduced in grains, suggesting that ZmG6PE may affect starch synthesis through the modulation of α-D-glucose-6-phosphate. β-D-glucose-6-phosphate participates in pentose phosphate metabolism to provide NADPH for life activities (Efferth et al., 2006; Mamani et al., 2016). Glycolysis and the pentose phosphate pathway occur in the cytoplasm, while the ZmG6PE protein is subcellularly localized in the nucleus and cell membrane. Omics analysis revealed significant enrichment of glycolysis/gluconeogenesis and pentose phosphate pathway-related processes. This suggests that ZmG6PE may indirectly participate in glycolysis and the pentose phosphate pathway by regulating the balance of glucose-6-phosphate. In addition, fructose-6-phosphate and mannose-6-phosphate may be potential substrates of glucose-6-phosphate-1 epimerase, indicating its broad substrate specificity (Graille et al., 2006). Significant differences were also observed in combined analysis for mannose-6-phosphate, indicating that mannose-6-phosphate may serve as a catalytic substrate for ZmG6PE, but further validation is required.

### 4.2 Sugars are key regulators for Pi starvation signaling at the transcriptional level

Sugars are primary metabolites and signaling molecules, and starch is the final product of photosynthesis and the dominant form of energy storage in plants. Phosphorus deficiency makes soluble sugar accumulation in leaves, while exogenously applied sugars can alleviate the phosphorus starvation response (Hermans et al., 2006; Yang et al., 2020). Similarly, LP treatment increases the starch content (Gibson, 2004; Morcuende et al., 2007). The results showed a cascade relationship between carbon metabolism and phosphorus signaling. However, the specific interaction between carbon metabolism and phosphorus remains unclear. ADP-glucosepyrophosphorylase (AGP) Large Subunit 1 (*AGPL1*) and AGP Small Subunit 1 (*AGPS1*) increases the starch and soluble sugar contents in leaves under LP, but the starch and sugar contents do not change significantly in the roots (Meng et al., 2020). The *zmg6pe* mutants had reduced soluble sugar content and increased starch content in the leaves under LP conditions but also had reduced starch content and increased sugar content in the grain. Sugar and starch are the main carbon sources in plants and keep a dynamic balance (Flütsch et al., 2020). Non-metabolizable sugar analogs do not affect the expression of *PSI* genes. Although sugar input appears to be downstream of initial Pi sensing, it is required to complete the *PSI* signaling pathway (Karthikeyan et al., 2007). The cellulose synthase-like family (*OsCSLF6*) gene mutation increases the sugar and phosphorus content, and the expression of sucrose synthase and sucrose transporter genes is also induced in rice (Jin et al., 2015). The overexpression of the sucrose transport (*AtSUC2*) gene promotes sucrose transport and accumulation and enhances *PSI* gene expression and Pi accumulation (Lei et al., 2011). Sucrose and auxin play differential roles in the developmental responses of ontogenetically distinct root traits during Pi deprivation (Jain et al., 2007). Therefore, it is crucial to investigate the potential mechanism of PSI gene expression changes mediated by glycometabolism-related genes.

### 4.3 Biological pathways involved in response to LP stress

KEGG enrichment analyses of the transcriptomic and metabolomic data showed that amino acids, starch and sugar synthesis, photosynthesis, plant pathogens, ABC transporters, brassinosteroids, plant hormones, and citric acid metabolism responded to LP stress. LP stress significantly increases amino acids in plants (Hernández et al., 2009; Tawaraya et al., 2014; Sung et al., 2015). Plants increase starch content and reduce photosynthesis to avoid anthocyanins accumulation in leaves in LP environments (Wissuwa et al., 2005; Plaxton and Tran, 2011; Pant et al., 2015). The current study analysis showed that LP affected carbon fixation in leaves while starch and sugar synthesis in roots. Phosphorus starvation is also associated with microbial interactions. Under LP conditions, plants establish a mutualistic symbiotic relationship with microorganisms to increase phosphorus acquisition (Smith and Smith, 2011; Oldroyd and Leyser, 2020). These microorganisms may be arbuscular mycorrhizal fungi or species of *Colletotrichum*. ABC transcription proteins are involved in both aluminum tolerance and the inhibition of local phosphate signaling pathways (Dong et al., 2017). Brassinosteroids and phytohormones respond to LP stress due to the regulation of local phosphorus signaling pathways by hormones interacting with different signals (Kobayashi et al., 2013). Overexpression of citrate synthase and transporter can improve the phosphorus utilization efficiency (PUE) by increasing organic acids secretion (López-Arredondo et al., 2014; Panchal et al., 2021). These pathways have all been reported in plant responses to LP stress. This evidence shows that the design of this research is reasonable and that these pathways are important in response to LP stress.

### 4.4 LP induces the expression of *ZmSPXs* and *ZmPHTs*

Phosphate Starvation Response (PHR) transcriptional factors are characterized by a conserved MYB domain and can participate in the transcriptional activation of *PSI* genes (Zhou et al., 2008; Guo et al., 2015). Phosphorus transport (PHT) regulates the uptake and transport of phosphorus (Paszkowski et al., 2002). SPX1 and SPX2 interact with PHR through their SPX domains, thereby inhibiting the binding ability of PHR to P1BS (Liu et al., 2010; Wang et al., 2014). Under LP stress, the expression of *ZmSPXs* increased, indicating that *ZmSPXs* inhibited *ZmPHR* and *ZmPHT* activities and affected the absorption and transport of phosphorus. Compared to the WT, the phosphorous content in *zmg6pe* mutant leaves decreased under NP conditions but remained significantly unchanged in *zmg6pe* mutant under LP stress conditions. Combined with the expression analysis, the expression of *ZmSPX2* decreased in the *zmg6pe* mutant under LP stress, indicating that *ZmG6PE* had a stimulative effect on *ZmSPX2*. The regulatory effect of ZmG6PE on ZmSPX2 may be achieved by influencing carbohydrate homeostasis or by modulating the expression of relevant genes in the nucleus. ZmG6PE might play a potential role in coordinating cellular metabolism and gene expression. The mutation of *AGPL1* and *AGPS1* lead to the significant downregulation of *OsSPX2* (Meng et al., 2020). AGPL1/AGPS1 is a starch synthesis enzyme, and ZmG6PE is an enzyme of the glycolysis pathway, suggesting that the carbon balance may affect SPX activity.

### 4.5 ZmG6PE regulates phosphorus transport and affects maize yield

**Figure 7:**
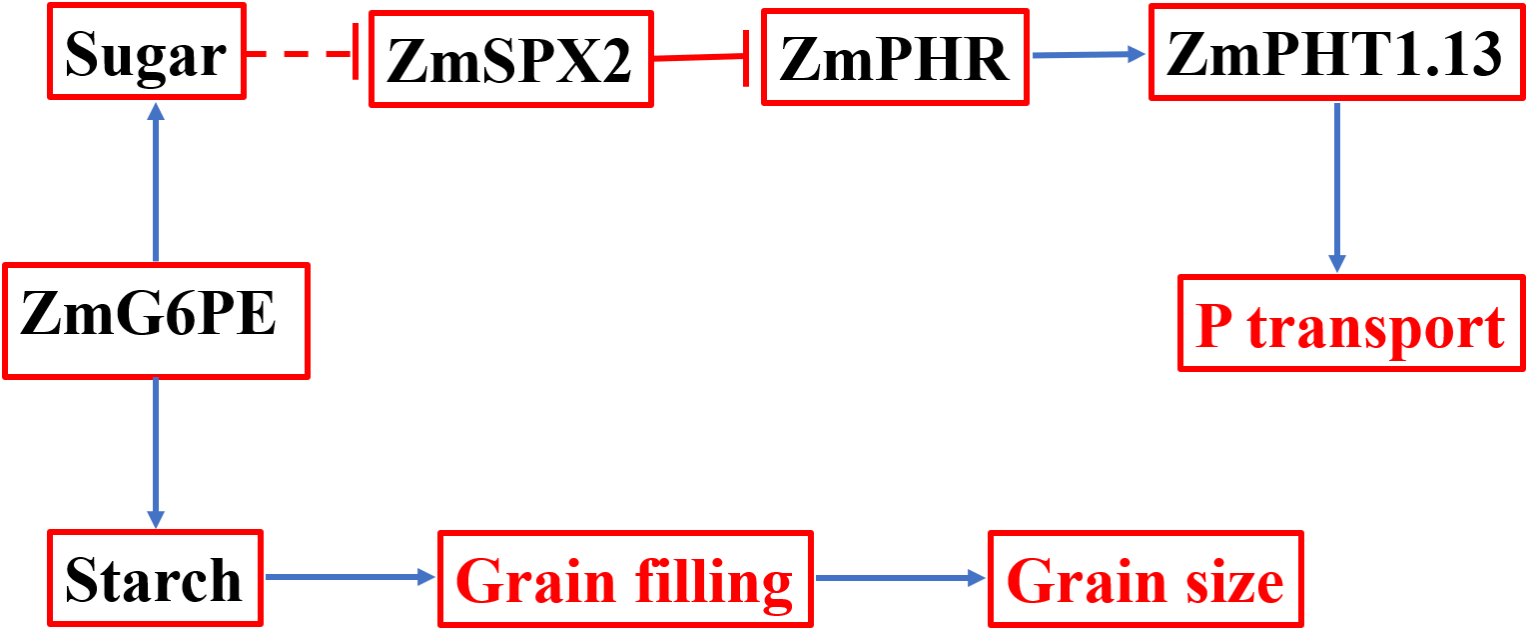
If working model for regulating phosphorus homeostasis by ZmG6PE. Solid lines indicate established direct correlations between different genes/proteins and between ZmG6PE, sugar, and starch. The dotted lines indicate indirect correlations between endogenous sugar level and SPX transcript abundance.

Based on the above findings, we put forward a working model involving *ZmG6PE* in phosphorus homeostasis maintenance in maize (**Figure 7**). ZmG6PE is attributed with the capability to modulate the dynamic equilibrium between two carbohydrate sugar and starch. The expression of *ZmSPX2* is subjected to regulation by sugar concentration, while it was found to have no discernible effect on *ZmPHRs* expression. Notably, despite a prominent decrease in *ZmPHT1.13* expression level observed in the *zmg6pe* mutant compared to WT, the unaltered expression of *ZmPHRs* appears to compensate for the deficit by modulating the expression of other *ZmPHTs*. This may account for the absence of significant variations in phosphorus levels under LP conditions between the *zmg6pe* mutant and WT. ZmG6PE is implicated in phosphate uptake through the regulation of *ZmSPX2* and *ZmPHT1.13* expression. Additionally, ZmG6PE exerts an influence on starch synthesis by modulating the α-D-glucose-6-phosphate content, consequently impacting grain filling and altering grain size.

## 5 Conclusion

The conserved domain of ZmG6PE protein ranges from amino acid 41 to 308, which is identified as the Aldose_epimase domain. It exhibits activity as a glucose-6-phosphate-1-epimerase and may possess substrate diversity. Omics analysis reveals that the *ZmG6PE* gene is involved in LP stress response through pathways, such as amino acid metabolism, carbon metabolism, genetic material biosynthesis, fatty acid metabolism, and immune regulation. Glucose-1-phosphate, mannose-6-phosphate, and β-alanine are identified as marker metabolites in response to LP stress. The *ZmG6PE* gene regulates the expression of *ZmSPX2* and *ZmPHT1.13* by modulating the dynamic balance of carbohydrates, thus participating in phosphate signaling regulation. Mutant studies have demonstrated the impact of ZmG6PE on starch synthesis, grain size, and yield-related traits, while the use of RILs has revealed its influence on yield-related traits and phosphorus content. The *ZmG6PE* gene is a pleiotropic gene that influences maize grain size and responds to LP stress.

## Data availability statement

Transcriptomic data has been submitted to the China National Centre for Bioinformatics, Chinese Academy of Sciences, under the accession number CRA010809, and is publicly accessible at https://ngdc.cncb.ac.cn/gsa.

## Ethics statement

The manuscript presents research on animals that do not require ethical approval for their study.

## Author contributions

HKZ: Writing – original draft, Writing – review & editing, Data curation, Validation. BWL: Writing – review & editing, Supervision. JL: Investigation, Writing – review & editing. XJ: Investigation, Writing – review & editing. HYZ: Data curation, Writing – review & editing. HXZ: Data curation, Writing – review & editing. BYL: Data curation, Writing – review & editing. HH: Data curation, Writing – review & editing. YW: Data curation, Writing – review & editing. AA: Formal Analysis, Writing – review & editing. AR: Formal Analysis, Writing – review & editing. JS: Formal Analysis, Writing – review & editing. MI: Formal Analysis, Writing – review & editing. XZ: Software, Writing – review & editing. DL: Supervision, Writing – review & editing. LW: Supervision, Writing – review & editing. DG: Resources, Writing – review & editing. SQG: Resources, Writing – review & editing. SS: Supervision, Writing – review & editing. SBG: Funding acquisition, Resources, Supervision, Writing – original draft, Writing – review & editing.
