# Supplementary material for: Functional analysis of ZmG6PE reveals its role in responses to low-phosphorus stress and regulation of grain yield in maize
Source: Front Plant Sci. 2023 Nov 9;14:1286699. doi: 10.3389/fpls.2023.1286699 (PMC10666784; doi:10.3389/fpls.2023.1286699)
Supplement: Supplementary file 1 [file DataSheet_1.docx]

**
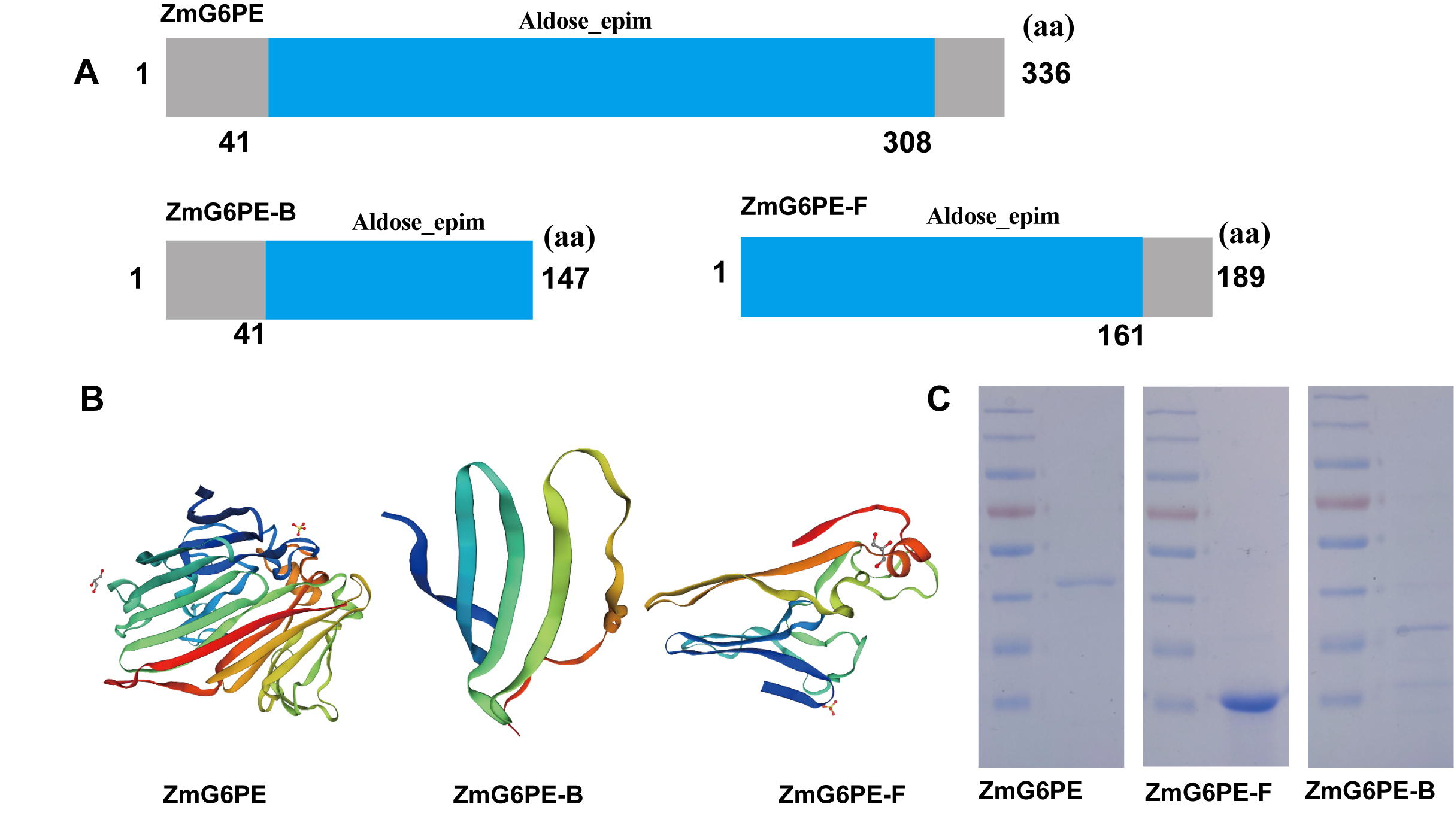
**

**Fig. S1.** Protein structure prediction and purification. (A) Conserved domain prediction of ZmG6PE, ZmG6PE-B, and ZmG6PE-F proteins. (B) The predicted tertiary structure of ZmG6PE, ZmG6PE-B, and ZmG6PE-F proteins. (C) Purification of ZmG6PE, ZmG6PE-B, and ZmG6PE-F proteins.

**
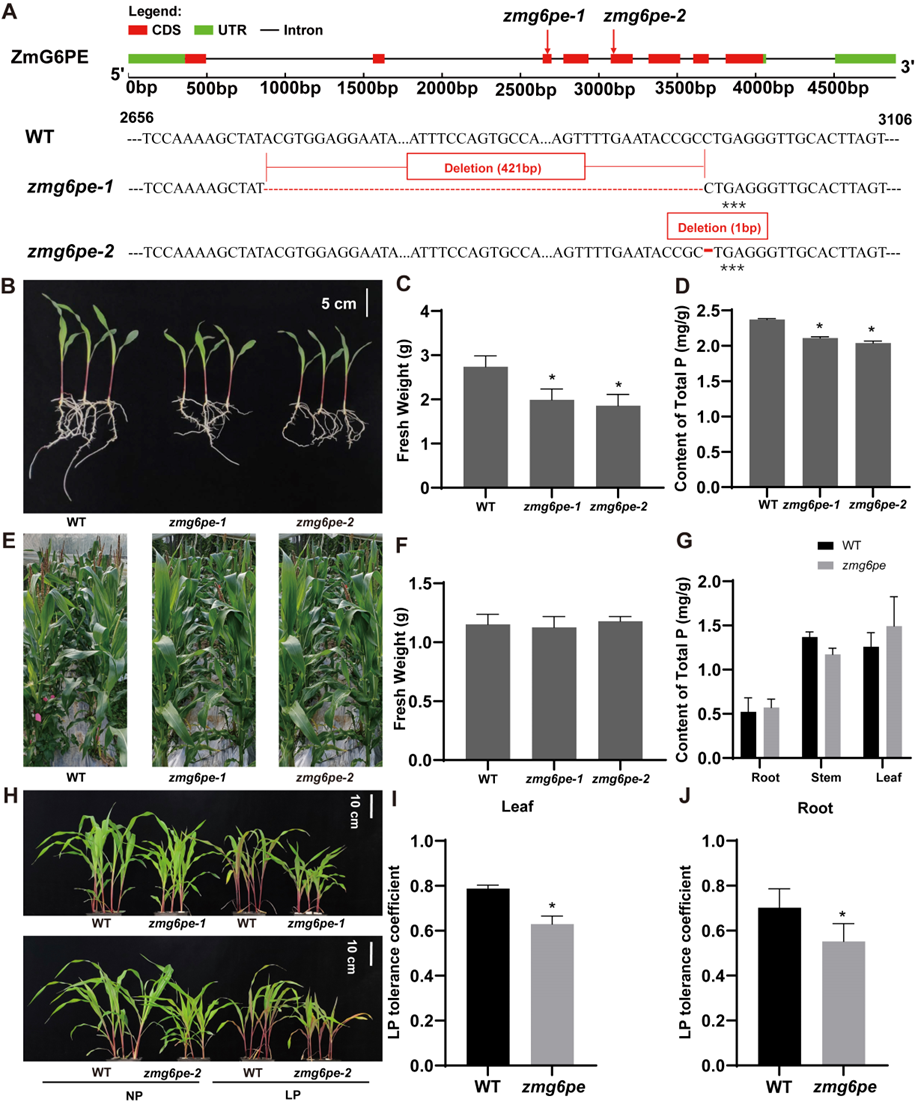
**

**Figure S2.** Mutant detection and analysis. (A) Determining the *ZmG6PE* gene mutation site. (B) Shoots of the WT and *zmg6pe*. (C) Fresh weight of the shoot. (D) Total P content of the shoot. (E) Maturity of the WT and *zmg6pe*. (F) Fresh weight of the WT and *zmg6pe* at maturity. (G) Total P of different tissues under field planting. (H) WT, *zmg6pe-1*, and *zmg6pe-2* mutants under NP and LP hydroponic conditions. (I–J) LP tolerance coefficient of leaves and roots in the WT and *zmg6pe* mutant, respectively. "*" represents p < 0.05 by single factor ANOVA test.

**
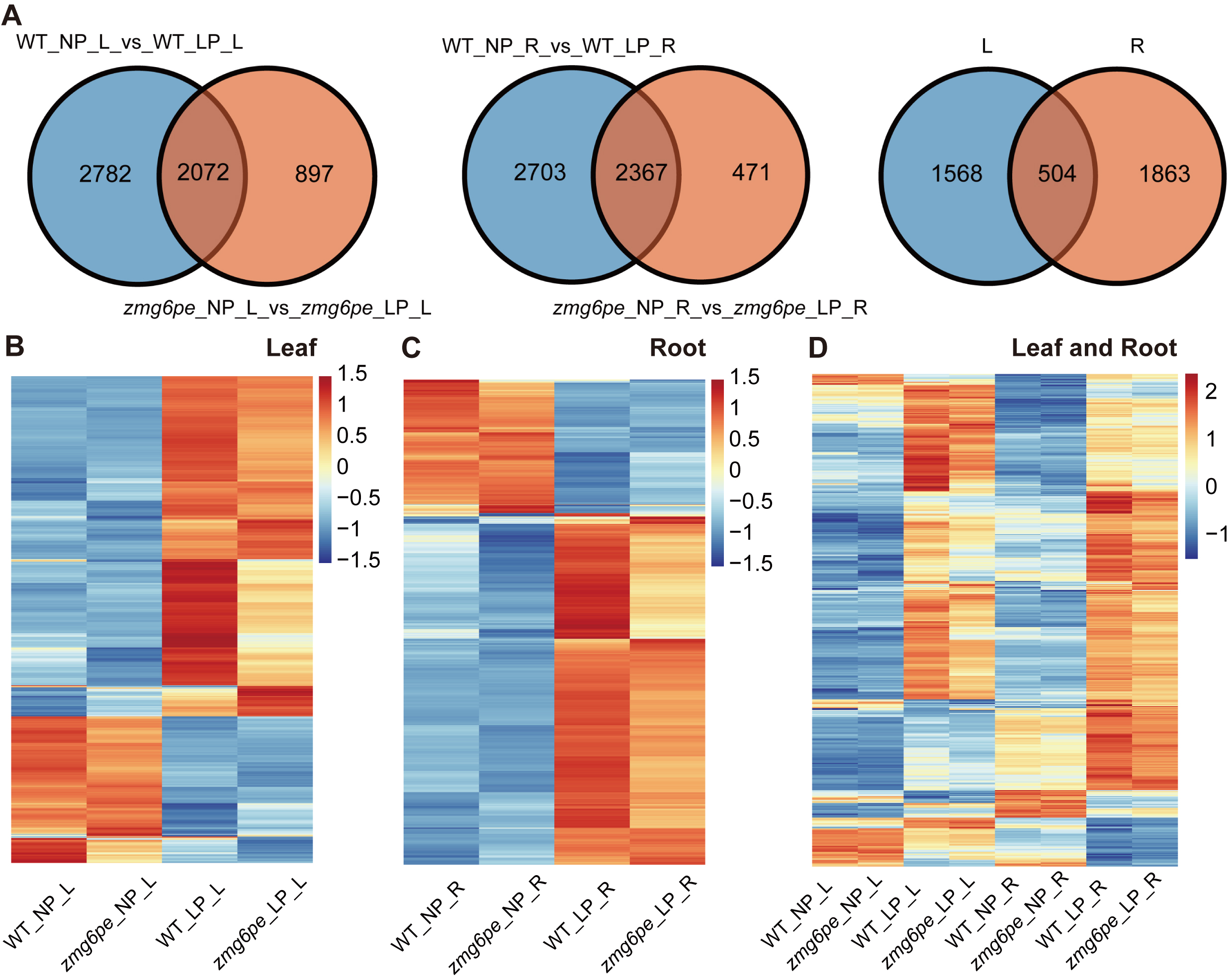
**

**Fig. S3.** A heatmap-based clustering analysis was conducted on the DEGs. (A) Venn diagrams showing the overlapping DEGs of leaves (left), roots (middle), and those shared in leaves and roots (right). (B–D) Heatmap showing the relative expression of 2072, 2367, and 504 overlapping DEGs in corresponding tissues, as shown above in leaves, roots, and shared in leaves and roots, respectively. Values are scaled by row.

**
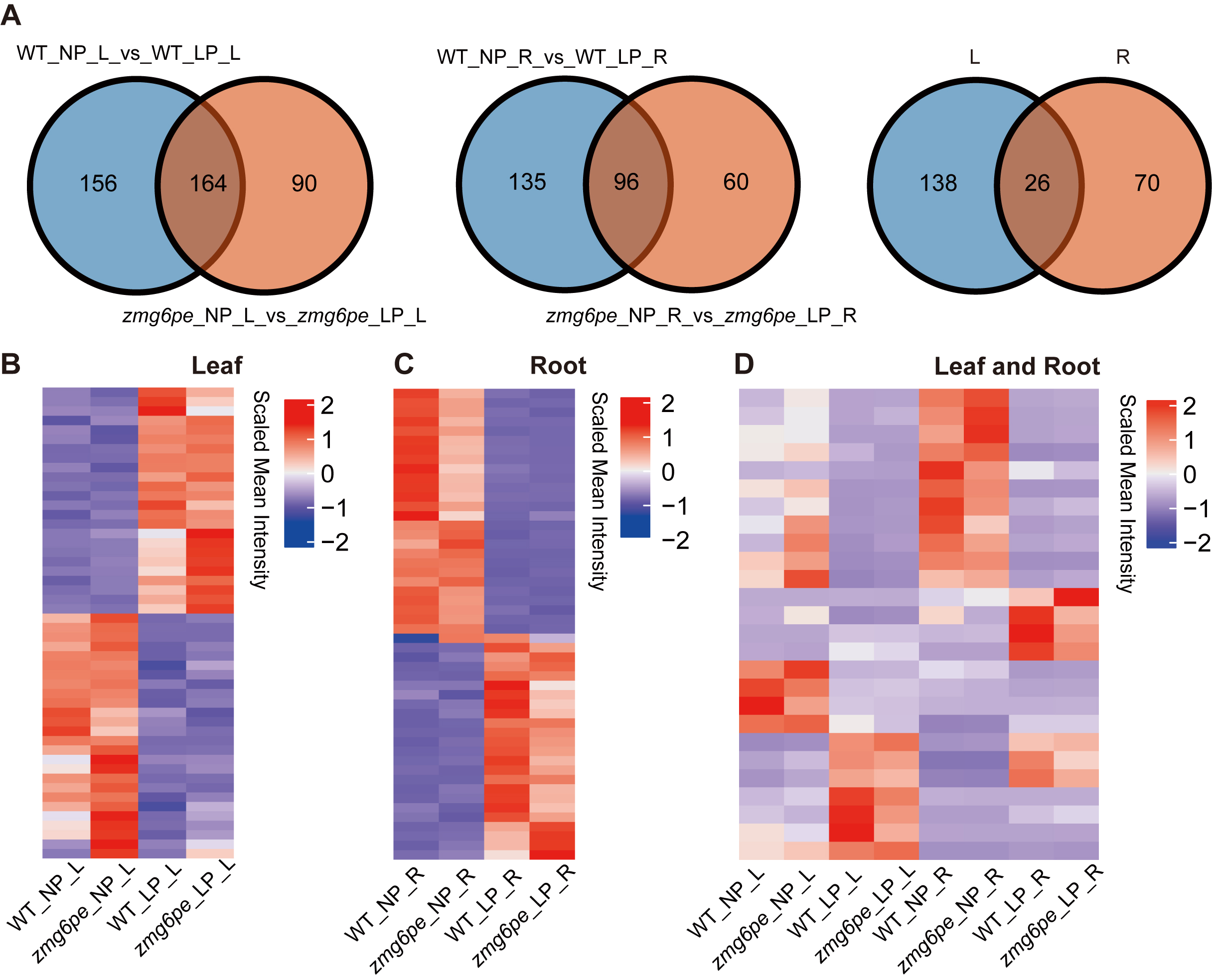
**

**Fig. S4.** A heatmap-based clustering analysis was conducted on the DEMs. (A) Venn diagrams showing the overlapping DEMs of leaves (left), roots (middle), and those shared in leaves and roots (right). (B–D) Heatmap showing the relative metabolite content of 164, 96, and 26 overlapping DEMs in corresponding tissues, as shown above in A. Values are scaled by row.


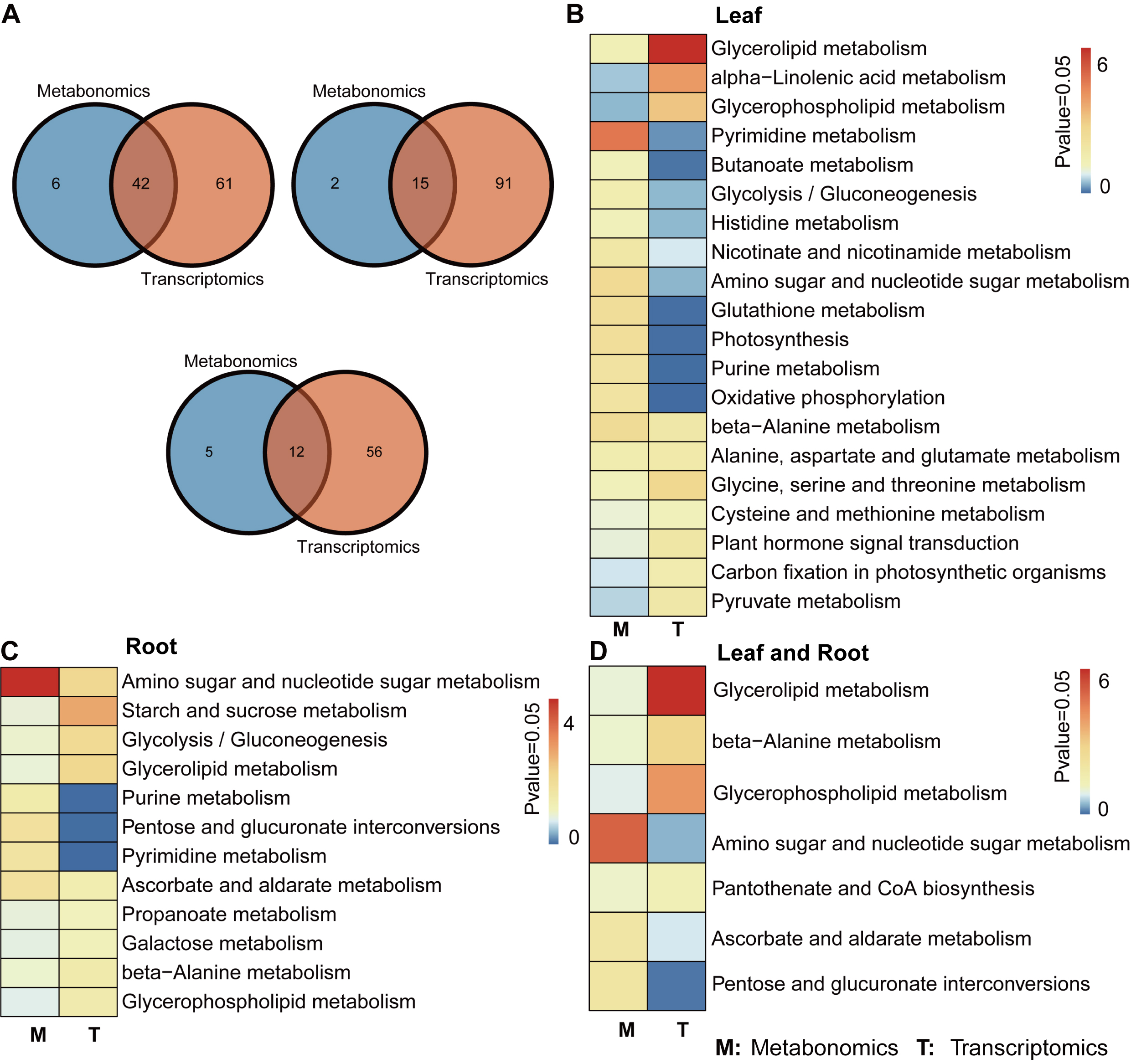


**Fig. S5.** A combined analysis of transcriptomic and metabolomic data. (A) Venn diagrams showing the transcriptome and metabolome of common KEGG pathways from leaves (upper left), roots (upper right), and those shared in leaves and roots (bottom). (B–D) Transcriptome and metabolomes of common KEGG pathways from leaves, roots, and those shared in leaves and roots, respectively.

**
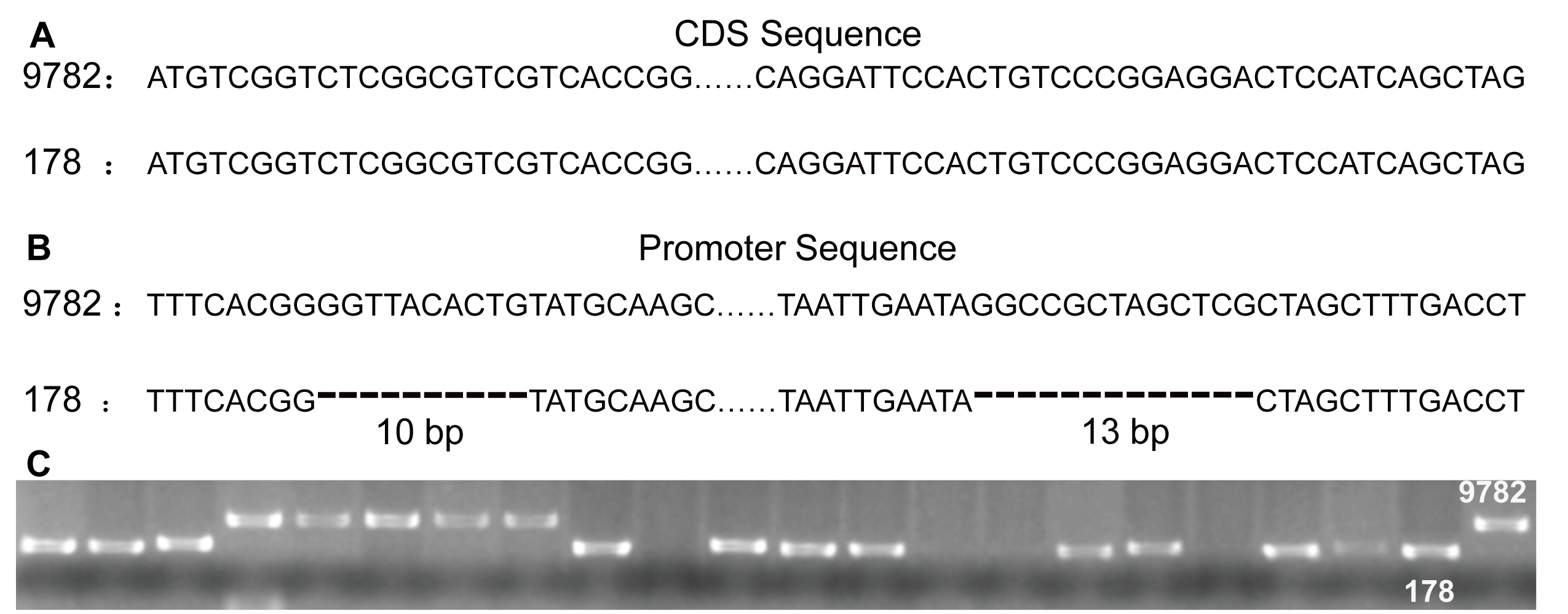
**

**Fig. S6.** Genotype verification. (A) CDS sequence alignment. (B) Promoter sequence alignment. (C) Verification of the RIL population by agarose gel electrophoresis.

**Table S1.** Examination of mutant and quantitative PCR

| **Classification** | **Primer name** | **Sequence** |
| --- | --- | --- |
|  | ZmG6PE-KO-F | 5’-TGTCCATCGAAAAGCAGCAC-3’ |
| Examination | ZmG6PE-KO-R | 5’-TCCTTCTACTCGCACCTCAC-3’ |
| of primer | ZmG6PE-InDel-F | 5’-AAACCAAAAGGCACGTTCCA-3’ |
|  | ZmG6PE-InDel-R | 5’-TGTCGACAGTTTCCTCAGGA-3’ |
| Quantitative PCR primer | ZmG6PE-qPCR-F | 5’-CAGTGCCAACCTCCAATAGC-3’ |
|  | ZmG6PE-qPCR-R | 5’-CCCTCAGGCGGTATTCAAAA-3’ |
|  | ZmPHT1.13-qPCR-F | 5’-CACCAGCTTCTTCGCCAATT-3’ |
|  | ZmPHT1.13-qPCR-R | 5’-AGCATGCTTCGGTTGTTGTT-3’ |
|  | ZmSPX2-qPCR-F | 5’-TCGACAAGTTCAACGCCTTC-3’ |
|  | ZmSPX2-qPCR-R | 5’-TCTCCAGCAAGACCATCTCG-3’ |

**Table S2.** Yield-related traits

| Year | Traits | Bald tip (mm) | Ear length (mm) | Ear thickness (mm) | Shaft thickness (mm) | 100-grain weight (g) | Ear weight (g) | Shaft weight (g) | Number of grains per ear |
| --- | --- | --- | --- | --- | --- | --- | --- | --- | --- |
| 2021-QJ | WT | 32.05±  2.22 | 103.53±  3.11 a | 40.21±  0.74 a | 29.92±  0.52 | 26.77±  0.79 a | 53.15±  3.77 a | 20.43±  0.79 a | 220.34±  23.46 a |
|  | *zmg6pe* | 30.07±  2.35 | 92.12±  1.82 b | 37.65±  0.53 b | 29.31±  0.41 | 20.42±  0.66 b | 38.80±  2.15 b | 18.61±  1.77 b | 104.41±  18.79 b |
| 2022- | WT | 12.05±  1.52 b | 135.1±  8.85 a | 39.11±  1.88 | 26.41±  1.89 | 18.79±  1.49 a | 76.59±  10.72 a | 20.10±  3.30 | 301.93±  48.92 a |
| CZ | *zmg6pe* | 18.47±  1.95 a | 115.1±  11.67 b | 38.25±  2.96 | 27.81±  2.88 | 15.80±  1.45 b | 49.57±  10.02 b | 21.90±  6.64 | 174.93±  37.46 b |

Qujing, QJ. Chongzhou, CZ. Different letters indicate significant differences, p < 0.05, single factor ANOVA test.
