# Supplementary material for: Functional analysis of ZmG6PE reveals its role in responses to low-phosphorus stress and regulation of grain yield in maize
Source: Front Plant Sci. 2023 Nov 9;14:1286699. doi: 10.3389/fpls.2023.1286699 (PMC10666784; doi:10.3389/fpls.2023.1286699)
Supplement: Supplementary file 10 [file Table_10.docx]

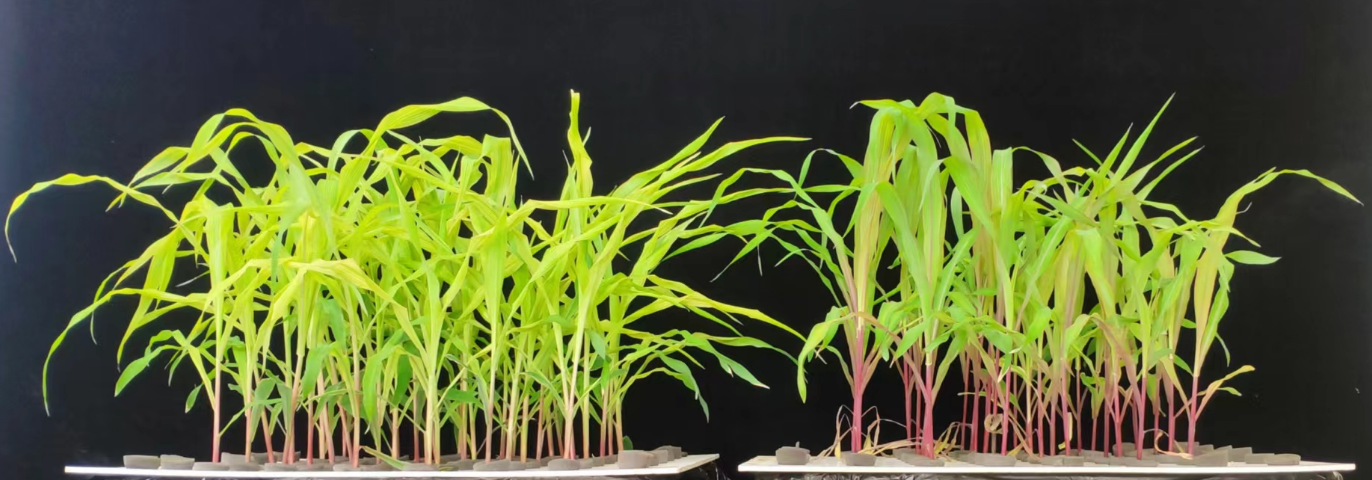


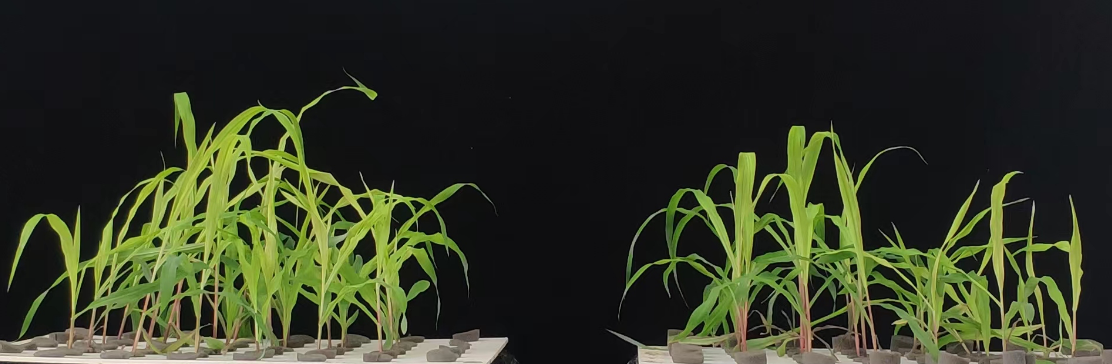


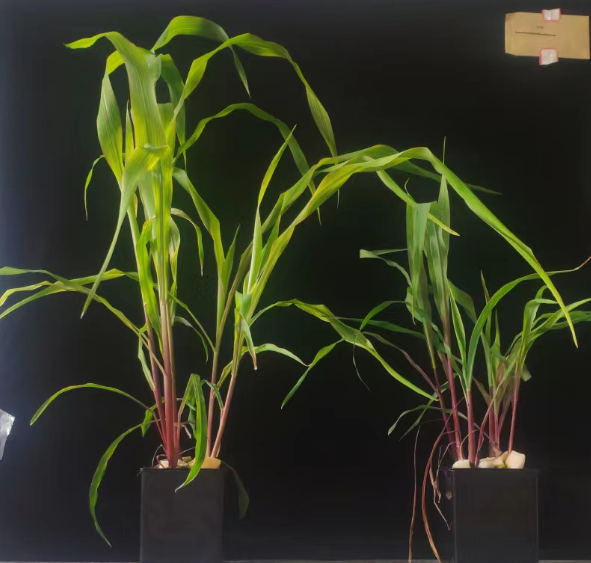


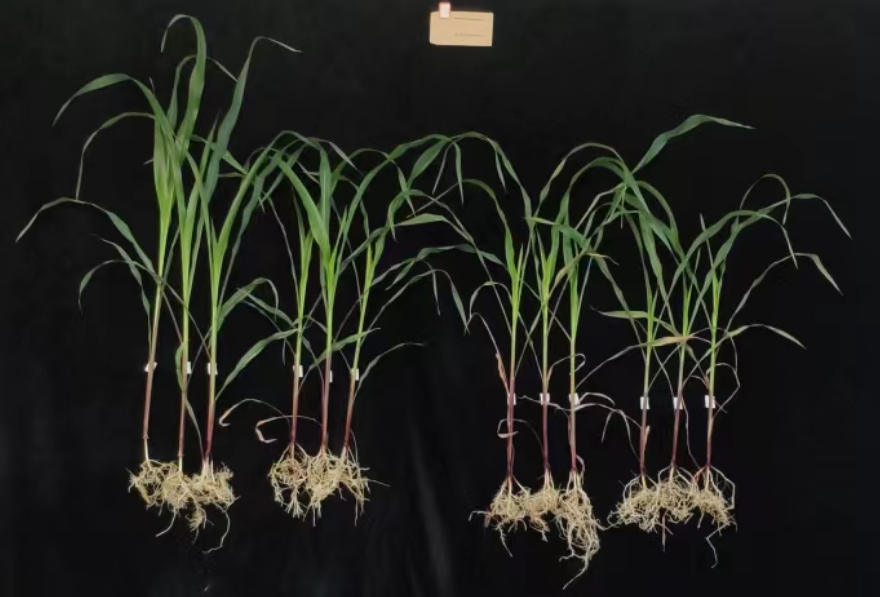


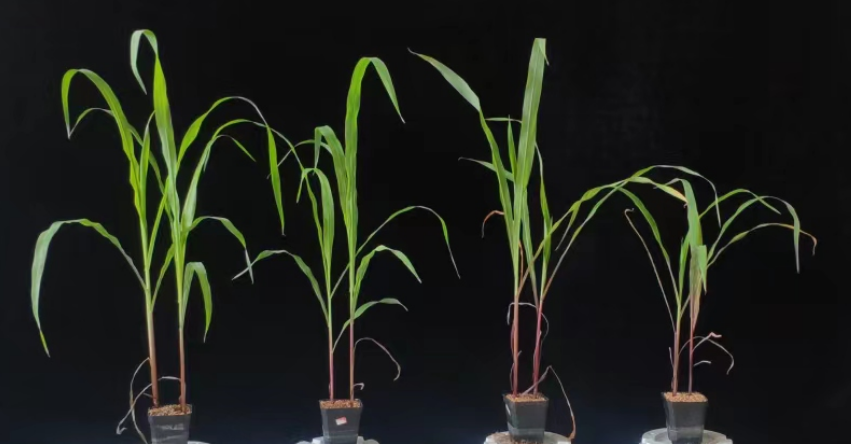


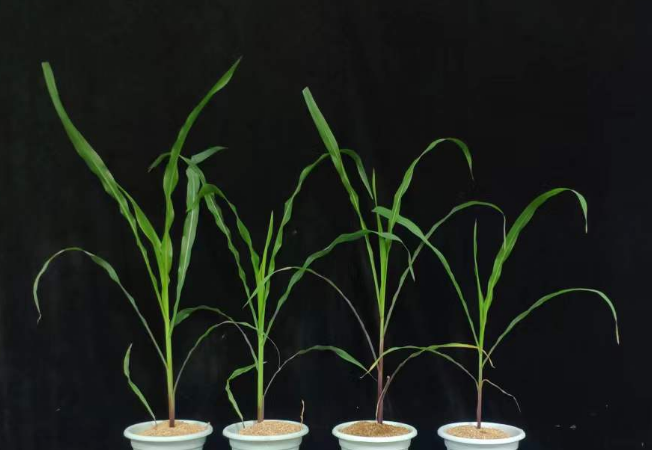


From left to right, they are WT-NP, zmg6pe-NP, WT-LP, and zmg6pe-LP.

Our experiments were repeated multiple times, and we consistently obtained the same results.
